# Evaluation of the Diabetes Health Plan to Improve Diabetes Care and Prevention

**DOI:** 10.5888/pcd10.120150

**Published:** 2013-01-31

**Authors:** O. Kenrik Duru, Carol M. Mangione, Charles Chan, Abigail Keckhafer, Lindsay Kimbro, K. Anya Kirvan, Norman Turk, Robert Luchs, Jinnan Li, Susan Ettner

**Affiliations:** Author Affiliations: Carol M. Mangione, Lindsay Kimbro, Norman Turk, Jinnan Li, Susan Ettner, David Geffen School of Medicine and Jonathan and Karin Fielding School of Public Health, University of California, Los Angeles; Charles Chan, Abigail Keckhafer, K. Anya Kirvan, Robert Luchs, Innovations Group, UnitedHealthcare, Minnetonka, Minnesota.

## Abstract

Investigators from the University of California, Los Angeles (UCLA), and members of the leadership and data analysis teams at UnitedHealthcare (UHC) are partnering to evaluate the Diabetes Health Plan (DHP), an innovative disease-specific insurance product designed by UHC specifically for patients with prediabetes or diabetes. The DHP provides improved access to care management, telephone coaching, and enhanced Internet-based communication with enrollees. The evaluation will use a quasi-experimental design, comparing patients from employer groups that offer the DHP with patients from groups that do not, to determine the effect of the DHP on incidence of diabetes, adherence to metformin, and costs of care among patients with prediabetes. Other factors studied will be cardiovascular risk factor control, adherence to preventive services, health care use, and costs of care among patients with existing diabetes.

## Introduction

Although diabetes incidence in the United States is rising for all ages, people aged 45 to 64 are most affected; diabetes incidence among people in this age group is 30% higher than among adults aged 65 or older (1). Many of these middle-aged patients are at risk of current and future disability from diabetes-related complications, and the combined direct and indirect costs of diabetes care for people in this group, including decreased productivity at work and increased absenteeism, are substantial (2). Public health stakeholders and employers share an interest in decreasing complications among patients with existing diabetes and in slowing disease progression among patients with prediabetes through early identification and increased adherence to preventive care and treatments. The Diabetes Health Plan (DHP), developed by UnitedHealthcare (UHC), is an innovative, multifaceted approach to prevent diabetes and improve diabetes management among working-age adults with the disease.

The DHP incorporates several enhancements to standard employer-based commercial benefit plans, including financial incentives of $150 to $500 per year for enrollees. These enhancements typically include reduced or eliminated copayments for office visits and for medications that reduce incidence of and complications from diabetes, access to diabetes-specific care management and individualized telephone coaching, enhanced Internet-based communication with beneficiaries via online data and adherence tracking, and improved access to diabetes education and information (3). Although participating employers are not required to offer each of these DHP components, more than 95% discount patient copayments for enrollees and most employers provide the other services. Results of some studies indicate that reducing copayments for evidence-based medications (ie, value-based insurance design) can modestly improve adherence to these medications (4,5), although these studies were not diabetes-specific and examined a single outcome (medication adherence) for follow-up periods of 12 months or less. The study we describe will be the first comprehensive, controlled, longitudinal evaluation to assess whether reduced cost-sharing for these services among people with prediabetes and diabetes improves multiple outcomes.

This real-time evaluation of the DHP is being conducted jointly by investigators at the University of California, Los Angeles (UCLA), together with employees of the Innovations Group at UHC, under the auspices of a cooperative agreement with the Centers for Disease Control and Prevention and the National Institute of Diabetes and Digestive and Kidney Diseases. Our collaboration is grounded in the principles of community-based participatory research (6,7) and builds on a long-standing partnership between the UCLA research team and the leadership and data analysis teams at UHC, 1 of the largest providers of health insurance in the United States. Our experience evaluating system-level interventions, analyzing health plan data, and disseminating findings tells us that these evaluations must be conducted in close partnership. The health plan side of the partnership brings intricate knowledge of employer group-specific aspects of program implementation, detailed knowledge about their data, and perspective on the interpretation of the results. The academic side of the partnership contributes state-of-the-art analytic modeling, grounds the hypotheses in findings from peer-reviewed scientific literature, leads an objective evaluation, and provides the needed policy context.

The design of the DHP was influenced by findings from the Translating Research into Action for Diabetes (TRIAD) study, which examined the effectiveness of care management (8) and the deterrent effect of high copayments on use of needed services and medications for people with diabetes (9,10). This partnered evaluation of the DHP is a logical next step in an innovative model of translational research over more than a decade (11). This study will provide evidence to determine whether a benefit design that is tailored to the needs of people with a specific condition and provides low cost-sharing will reduce incidence of diabetes among people with prediabetes and improve cardiovascular risk factors, reduce complications, and lower costs for patients with diabetes.

This study is being conducted in a real-world setting, similar to the way many new health insurance products are implemented in employer groups. The quasi-experimental design fits well into the framework of the Natural Experiments in Translation for Diabetes (NEXT-D) study’s goals of evaluating natural experiments by using rigorous statistical methods. The findings from this study will provide useful information to employers, health plans, and public health stakeholders about the effectiveness of this type of multifaceted diabetes care and prevention approach.

## Study Population, Study Design, and Analytic Approach

The DHP was first introduced in 2009 and has been purchased by more than 2 dozen national and regional employers who contract with UHC. These employers represent a diverse spectrum, including industrial and manufacturing companies, service and retail companies, and public sector organizations. The DHP is an available option for employees and in most cases their spouses and immediate family members who are identified as having prediabetes or diabetes on the basis of laboratory history, diagnosis codes from medical claims, results of employer-based biometric screenings, or physician verification of the diagnosis. Although most beneficiaries in the DHP can maintain program benefits each year without conditions, several participating employers require adherence to “compliance criteria.” These criteria require recommended diabetes and nondiabetes preventive care (eg, hemoglobin A1c and cholesterol screening, retinal eye exams, mammograms for women aged 40 or older) to maintain ongoing enrollment.

This study will test several hypothesized effects of the DHP. We hypothesize that among patients with prediabetes, those insured through employers offering the DHP will have greater initiation of and adherence to metformin for diabetes prevention, less progression to diabetes over a 3-year period, and lower costs of care than for comparable patients with prediabetes insured through employers that do not offer the DHP. We hypothesize that patients with diabetes who are insured through employers offering the DHP will have better glycemic and lipid control, better adherence to diabetes-specific and general preventive services, less emergency department use, fewer hospitalizations, and lower total costs than patients insured through employers that do not offer the DHP.

This study will treat the DHP as a “natural experiment” because the program is not a true research-designed experiment. However, the DHP represents a clearly defined set of new benefits provided by certain employers for their patients with diabetes and prediabetes. Therefore, changes in prespecified study outcomes among these patients, compared with outcomes for similar patients from other employer groups with an unchanging benefit structure, can be reasonably attributed to the effect of the DHP.

Using a quasi-experimental design to test our hypotheses is equivalent to comparing the change in outcomes over time among patients from employers that offer the DHP with changes among concurrent patients from employer groups that offered UHC insurance products other than the DHP. Unlike a simple pre-post comparison, our use of a comparison group allows us to adjust for the effects of secular time trends over a 3-year period; in turn, unlike a simple cross-sectional comparison, our use of longitudinal data allows us to adjust for any baseline differences between the DHP and non-DHP groups that may confound the comparison. The “difference in differences” analyses for each outcome (eg, number of diabetes-related hospitalizations among patients with existing diabetes), will be structured as follows: [(mean difference for patients of DHP employers expressed as follow-up minus baseline) – (mean difference for patients of non-DHP employers expressed as follow-up minus baseline)]. Although our quasi-experimental design requires an assumption that the secular time trends are similar for the DHP and non-DHP groups, we can test this assumption in cases in which multiple years of existing data are available for both the DHP and non-DHP groups. Because DHP enrollment may change from year to year depending on whether or not enrollees meet the compliance criteria and whether their employer continues to purchase the DHP from UHC, we will include the DHP as a time-varying covariate.

## Dissemination of Study Findings and Potential Effect

The DHP incorporates a value-based structure in which patient cost-sharing is reduced for evidence-based therapies (eg, metformin to prevent diabetes, statins to prevent myocardial infarction). Millions of Americans will be exposed to value-based insurance products in the coming years, and results from this study will provide important information for patients deciding between health insurance options. In a 2011 survey, more than half of employers were considering adding a value-based insurance option in the next 3 to 5 years (12). Furthermore, section 2713 of the Affordable Care Act allows the US Department of Health and Human Services to establish guidelines for value-based insurance designs, including plans to be offered in health insurance exchanges (13).

The research team plans to publish in the peer-reviewed literature but will also write articles for trade journals read by health plan stakeholders and op-ed articles in mainstream media to publicize the findings for a nonacademic audience. Finally, the research team plans to produce user-friendly print materials for employees who are enrolled in DHP programs. The materials will be delivered with an eighth-grade literacy level or lower and will focus on explaining the specifics of the DHP in combination with information on the study findings.
